# Uncovering intersectional inequalities in fruit and vegetable consumption in the UK using Understanding Society data

**DOI:** 10.1017/S1368980026102328

**Published:** 2026-03-26

**Authors:** Patricia Stehl, Nina Vyvcharuk, Naomi Daniel, Neža Grilc, Armelle Müller, Sanne Verra

**Affiliations:** 1 University of Vienna, Austria; 2 Department of Methodology and Statistics, Tilburg University, Tilburg, Netherlands, Netherlands; 3 Harvard University, USA; 4 Brunel University London, UK; 5 Ludwig-Maximilians-Universität München, Germany; 6 Interdisciplinary Social Science, Utrecht University, Netherlands

**Keywords:** fruit and vegetable consumption, intersectionality, social inequality, diet, MAIHDA

## Abstract

**Objective:**

Large inequalities in fruit and vegetable consumption (FVC) persist, yet it remains unclear how intersecting factors such as socio-economic status, ethnicity and sex influence FVC in the UK. Using an intersectional framework allows us to explore complex realities and double burdens faced by certain population groups.

**Design:**

Cross-sectional data from the UK Household Longitudinal Study Wave 9 (2017–2018) were analyzed. FVC was measured as a binary variable, indicating whether individuals met the recommended five daily portions of fruits and vegetables (400 grams in total). An intersectional Multilevel Analysis of Individual Heterogeneity and Discriminatory Accuracy was used, nesting participants into forty-eight social strata based on sex, ethnicity, age and educational level.

**Setting:**

United Kingdom.

**Participants:**

A total of 16 275 individuals from the UK Household Longitudinal Study sample were included, with one adult randomly selected per household.

**Results:**

Overall, 69·2 % of the sample did not meet the recommended daily FVC. Inequalities were predominantly explained by additive effects of sex, ethnicity, age and educational level. Men, individuals with lower educational levels, ethnic minority groups and younger participants were at higher risk of insufficient FVC, particularly those experiencing combinations of these factors.

**Conclusions:**

Low FVC across the population, combined with strong additive effects of social determinants, underscore the need for proportionate universal interventions. Policies targeting improved access to fruits and vegetables across all neighbourhoods, especially those predominantly inhabited by individuals with lower educational levels, are warranted to reduce these inequalities.

Healthy food consumption plays a crucial role in preventing chronic diseases and promoting overall wellbeing. Dietary patterns identified in the Global Burden of Disease studies as ‘healthy’ are those that minimize the risk of CVD, diabetes and cancer.^(1)^ Healthy diets are typically rich in plant-based foods and low in added sugars, Na and unhealthy fats. Consistent with this, the UK’s ‘*5 A Day’* campaign, launched nationally in the UK in 2003, recommends the consumption of at least five portions (400 grams) of fruits and vegetables daily.^(2)^

However, despite the awareness of the universal health benefits of healthy food consumption, adherence remains low. Unhealthy diets high in processed meats, refined grains, sugar-sweetened beverages, Na and foods high in saturated and trans-fats remain common worldwide. In the United Kingdom, adherence to dietary guidelines is notably poor, particularly fruit and vegetable consumption (FVC): only an estimated 20 % to 27 % of the UK population meets the recommended intake of FVC.^(3,4)^ If the entire UK population adhered to the FVC recommendations, approximately 15 000 premature deaths could be prevented each year.^(5)^

While ongoing efforts to promote healthy eating behaviour across the UK^(6,7)^ have led to some improvements,^(8)^ large inequalities in FVC persist.^(4,9)^ People with a lower socio-economic position (SEP) are much less likely to meet FVC recommendations.^(4)^ Worldwide, as well as in the UK, educational attainment specifically has been identified as one of the most significant predictors of FVC,^(10,11)^ and individuals with a lower educational attainment level tend to have lower FVC than their more educated counterparts.^(10–16)^ Evidence on ethnic differences in FVC is less clear. While some studies report higher FVC among ethnic minority groups compared with ethnic majority groups,^(4)^ others highlight systemic and structural barriers that may limit access to healthy diets among ethnic minority populations in the UK.^(17)^ The evidence regarding sex differences is inconclusive, with some studies reporting a lower FVC among males than females,^(11,13)^ and other studies indicating no difference.^(4)^ FVC also varies by age and is particularly low among young adults and elderly people.^(4,18,19)^

Although many studies have examined how specific mechanisms of inequality in, e.g. SEP, ethnicity and sex, shape one’s FVC in isolation, in reality, these mechanisms operate simultaneously.^(20)^ It is not fully understood how inequalities jointly operate in their influence on FVC in the UK.^(4)^ Intersectionality theory, developed by critical race theorist Kimberlé Crenshaw,^(20)^ posits that the various elements of a person’s identity intersect, and that the study of these intersecting variables could reveal deprivations that are greater than the sum of their parts.^(21,22)^ By examining inequalities in FVC across multiple intersections of (dis)advantage, we can explore if some population groups face systemic, structural barriers to healthy FVC, such as limited availability and accessibility or stigma.^(4,23)^ Analysing how people’s shared characteristics may impact one’s FVC allows us to better capture the complex realities and burdens some population groups may face in their health behaviour.

Intersectionality theory is increasingly applied to understand joint health inequalities.^(24)^ Yet, to date, few intersectional studies have examined health in the context of the UK.^(24,25)^ Intersectional inequalities in FVC have been examined among the Swedish population based on gender and educational level. That study showed that being male with low education was associated with lower FVC, and being female with high education was associated with a higher FVC.^(26)^ To our knowledge, only one study has examined FVC in the UK through an intersectional lens. That study was conducted among older populations in the UK and found that older men with a lower educational level and low economic resources had a less varied FVC than their female counterparts.^(12)^

The current study aims to gain a deeper understanding of intersectional inequalities in FVC based on educational level, sex, age and ethnicity among adults in the UK. Improving our understanding of which populations may be at risk of unhealthy FVC will allow policymakers to address potential structural barriers these populations may face in obtaining their FVC and could inform targeted interventions to mitigate these inequalities.

## Methods

### Sample

Data from Understanding Society, the UK Household Longitudinal Study, a nationally representative survey comprising approximately 26 000 households in the UK was used.^(27)^ The cross-sectional adult main interview from Wave 9 (2017–2018) was used, which was the most recent of the fourteen waves available at the time the study was conducted and included over 36 000 participants between the ages of 16 and 103.

**Figure 1.**
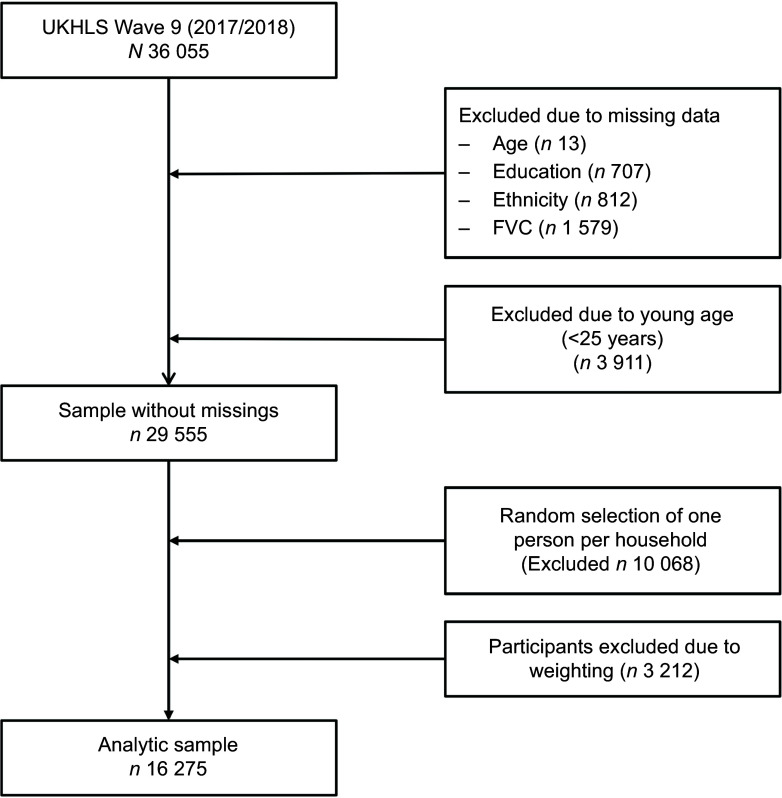
Flow chart indicating exclusion criteria and numbers of participants excluded from the analyses.

Figure 1 provides a flow chart of the sample used for analyses. In the first step, participants with missing values for one or more of the variables included in this study were excluded using listwise deletion. Next, to ensure every participant in our sample was likely to have completed their education, only participants aged 25 yearsor older were selected, resulting in the removal of 3991 participants. To resolve the issue of individuals being nested in households, we randomly selected one person from each household for our analyses, following the example of Begum & Sultana^(28)^ and Kline et al.,^(29)^ removing 10 068 participants from our sample. To ensure our random sub-sample was cross-sectionally representative of the population of the UK at the time of collection, the cross-sectional adult main interview sampling weights were applied. Since some demographic groups were over-represented, some participants were assigned a weight of zero, which led to the exclusion of 3212 overrepresented participants from the weighted analyses. The final representative sample consisted of 16 275 individuals. Ethics approval for the study was granted by the ethics committee of the Faculty of Social and Behavioural Sciences, Utrecht University (FSW FETC 22–211).

### Measures

#### Fruit and vegetable consumption

Since the UK’s ‘*5 A Day’* guidelines refer to a combined consumption of fruits and vegetables, a single measure of FVC, indicating whether participants met the recommended intake, was constructed. FVC was assessed with four questions. Participants separately reported the number of portions of fruits and the number of vegetables consumed per day. A portion of fruit was explained as one larger fruit or several smaller ones (e.g. an apple or some grapes), while a portion of vegetables was defined as three heaped tablespoons. Secondly, the number of days per week on which these products were consumed was assessed with the options ‘Never’, ‘1–3 d’, ‘4–6 d’ and ‘Every day’. These options were recoded into 0, 2, 5 and 7 d, respectively. For both fruit and vegetables, a daily average intake was calculated by multiplying the number of portions consumed per day by the number of days per week the participant reported eating that product, then dividing by seven. This provided one daily average for fruit and one for vegetables. These two daily averages were then summed to produce each participant’s combined daily mean FVC. Finally, this combined mean value was used to determine whether the participant met the recommended guideline. The mean variable was transformed into a binary outcome variable, classifying participants into either meeting (1) or not meeting (0) the recommended daily FVC.

#### Intersectional groups by educational level, sex, ethnicity and age

Four dimensions of socio-demographic variables were used to construct people’s intersectional positions: educational level, sex, ethnic group and age. Educational level was measured by the participant’s highest educational qualification. Research investigating health inequalities differs considerably in how educational attainment is coded and reported. The Understanding Society dataset provides six options, i.e. (1) Degree; (2) Other higher degree; (3) A levels; (4) General Certificate of Secondary Education; (5) Other qualification and (6) No qualification, which some studies condense to four levels.^(30,31)^ The same approach was used in the present study, and the categories were coded as follows: a higher education degree (e.g. university degree), secondary education qualifications (equivalent to UK A-levels or lower), no qualification or other qualification (e.g. professional or vocational qualifications). Sex was measured on a binary scale as male and female. These labels were used in our analysis. To measure ethnicity, the survey distinguished between eighteen different ethnicities. Ethnicity was used as a binary variable in this study due to insufficient sample sizes within ethnic minority groups. Participants were assigned to either the ethnic majority or ethnic minority group: ‘British/English/Scottish/Welsh/Northern Irish’, ‘Irish’, ‘Gypsy or Irish Traveller’ and ‘Any other white background’ made up the ethnic majority group in the present study, and ‘White and Black Caribbean’, ‘White and Black African’, ‘White and Asian’, ‘Any other mixed background, ‘Indian’, ‘Pakistani’, ‘Bangladeshi’, ‘Chinese’, ‘Any other Asian background’, ‘Caribbean’, ‘African’, ‘Any other Black background’ and ‘Arab’ made up the ethnic minority group. The age range in our sample was 25 to 103 years and was divided into three categories: 25–40 years, 41–60 years and 61 years and older.

The intersectional social strata were created by multiplying the number of categories across these variables (4 × 2 × 2 × 3), which led to forty-eight distinct intersectional strata. Each participant in the dataset was assigned to one of these forty-eight strata based on their observed combination of characteristics.

### Data analysis

Multilevel Analysis of Individual Heterogeneity and Discriminatory Accuracy (MAIHDA) was used to explore intersectional inequalities in FVC. This method is currently considered the ‘golden standard’ in quantitative intersectionality analyses.^(32–35)^ The approach differs from typical multilevel approaches in the sense that it does not cluster participants by an observable context like schools, but by people’s shared combination of the socio-demographic factors age, sex, ethnic group and educational level. Those combinations are called social strata. A 2-level model was used, in which individuals (Level 1) were nested within their respective social stratum (Level 2). FVC was assessed using multilevel logistic regression models, estimated at 95 % credible intervals (CI).

Following the approach by Evans et al.,^(36)^ the first model was an unadjusted random intercepts model, in which individuals are nested within their social strata. This model was used to estimate the total variance between the two levels. Based on this model, an initial intra class correlation (ICC), a measure of discriminatory accuracy, was calculated. The ICC indicates the share of the total variance that was located at the stratum level.^(35)^ The ICC provided an estimate of the relative importance of the strata; the higher the ICC, the greater the similarity of individuals’ FVC within each intersectional stratum. The ICC was computed as 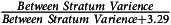 , where 3·29 denotes the within-stratum between-individual variance constrained to be equal to the variance of the standard logistic distribution.^(37)^ In line with Fisk et al.,^(38)^ the scale for psychometric tests was used to interpret the strength of the ICC values using the following cut-offs: non-existent (0–1), poor (> 1 to ≤ 5), fair (> 5 to ≤ 10), good (> 10 to ≤ 20), very good (> 20 to ≤ 30) and excellent (> 30). Additionally, the unadjusted random intercepts first model allowed us to calculate a predicted incidence for meeting the recommended FVC and the 95 % CI for every intersectional stratum. As a second measure of discriminatory accuracy, AUC statistic was reported. The AUC takes a value between 0·5 and 1·0 and indicates the probability that a randomly selected participant, who meets the recommended FVC, belongs to a stratum with a higher prevalence of meeting the recommended FVC compared with a randomly selected participant who does not meet the recommended FVC.

In the next step, an intersectional interaction model was fitted, where all variables that made up the intersectional strata were added as fixed main effects. While the ICC of Model 1 informed us about the maximum explanatory power of the strata, the ICC of Model 2 informed us about the part of Model 1 stratum variance that was due to the intersectional interaction effects on the stratum level. For example, if the ICC of model 2 indicates a high discriminatory accuracy, it implies that there are meaningful intersectional differences between the strata. This fully adjusted model also provided estimated residuals and their CI for every intersectional stratum. The models were fitted using maximum likelihood estimation in Stata version 16.

## Results

The sample was largely composed of individuals from the ethnic majority population (93·8 %) and included participants across all age groups. Women were somewhat overrepresented compared with men. Educational levels in the sample were generally high, with most participants reporting A-level or degree-level qualifications. The characteristics of the study population are presented in Table 1. Overall, 30·8 % of the sample met the recommended amount of daily FVC. Table 2 provides an overview of the two MAIHDA models with their respective ICC, OR and CI. Based on the OR from Table 2 (Model 2), females have higher odds of meeting the recommended FVC compared with males (OR: 1·58, 95 % CI: 1·42-1·77), and people belonging to ethnic minority groups had lower odds of meeting FVC recommendations compared with the ethnic majority (OR: 0·79, 95 % CI: 0·73-0·86). Middle-aged and older individuals have higher odds of meeting FVC recommendations than younger individuals (OR: 1·30, 95 % CI: 1·13-1·51 for middle age; OR: 1·78, 95 % CI: 1·53-2·06 for older age), and individuals with an education level below a degree have lower odds of meeting FVC recommendations than participants with a degree (OR: 0·56, 95 % CI: 0·49-0·65 for A-Levels; OR: 0·36, 95 % CI: 0·30-0·42 for no qualification; and OR: 0·48, 95 % CI: 0·39-0·56 for other qualification, all compared with people with a degree).

**Table 1.** Descriptive characteristics for socio-demographic variables and FVC

	*N* (%)
Fruit and vegetable consumption	
Meeting the recommendation	30·8 %
Not meeting the recommendation	69·2 %
Education level	
Higher education degree	39·8 %
A-levels	37·1 %
No qualification	12·4 %
Other qualification	10·7 %
Sex	
Male	43·7 %
Female	56·3 %
Ethnicity	
Ethnic majority	93·8 %
Ethnic minority	6·2 %
Age	
25–40 years old	23·5 %
41–60 years old	38·5 %
61 years old and older	38·0 %

FVC, fruit and vegetable consumption.

**Table 2.** Results from MAIHDA for adults in the UK in 2018. Model 1 is a random intercepts model with individuals nested within their social strata. Model 2 is fully adjusted, with all social dimensions being included as fixed main effects. Only the associations between the individual social dimensions and FVC are presented here

	Model 1		Model 2	
	OR	95 % CI	OR	95 % CI
**Fixed Effects: regression coefficients**				
Intercept	0·34	0·30–0·38	0·37	0·32–0·43
Sex				
Male (Ref)			–	
Female			1·58	1·42–1·77
Ethnicity				
Ethnic majority (Ref)			–	
Ethnic minority			0·79	0·73–0·86
Age				
25–40 years (Ref)			–	
41–60 years			1·30	1·13–1·51
60+ years			1·78	1·53–2·06
Qualification				
Higher degree (Ref)			–	
A-Levels			0·56	0·49–0·65
No qualification			0·36	0·30–0·42
Other qualification			0·48	0·39–0·56
**Random effects: variances**				
Stratum-level	0·24	0·14–0·38	0·01	<0·01–0·04
**Summary statistics**				
Intra class correlation (ICC; 95 % credible interval)	6·7 %	4·05–10·40	0·4 %	0·11–1·25
Proportional change in variance (PVC)			93·8 %	
Area under receiver operating characteristic curve (AUC)	0·63		0·61	

FVC, fruit and vegetable consumption.

The ICC of Model 1 (6·7 %, see Table 2) was assessed as fair, meaning that at least some share of the total individual differences in the likelihood of meeting the recommended FVC was located at the intersectional strata level. In the intersectional interaction Model 2 (Table 2), in which all social strata were added as fixed effects at the same time, the ICC dropped to 0·4 %, implying that inequalities in FVC were mainly driven by additive effects. According to Model 2, no social strata experienced intersectional interaction effects.

Table 3 displays the five intersectional strata with the lowest and highest predicted percentages of meeting the recommended FVC. The stratum with the highest predicted share of participants not meeting the recommended FVC consisted of young ethnic minority females with no qualification (9·4 %, 95 % CI: 7·0, 12·6), closely followed by young ethnic majority males with no qualification (9·4 %, 95 % CI: 6·7, 12·6). In contrast, the stratum with the highest predicted share of meeting the recommended intake consists of older-aged ethnic majority females with a degree (40·2 %, 95 % CI: 36·7, 43·8) followed by older ethnic majority males with a degree (39·3 %, 95 % CI: 36·2, 42·1). Even though there are considerable differences in FVC between the strata, Model 2 indicates that the intersectional interactions of the social dimensions do not significantly explain the variance in FVC.

**Table 3.** Predicted incidence for sufficient fruit and vegetable consumption for adults in the UK in 2018 by intersectional strata. Based on model 1 multilevel regression analysis with participants at the first level and intersectional strata at the second level

Rank	Sex	Ethnicity	Age	Education	*n*	Predicted % meeting FVC recommendation	Approximate 95 % CI
**5 lowest**							
1	Female	Ethnic minority	25–40	No qualification	26	9·4	7·0–12·6
2	Male	Ethnic minority	25–40	No qualification	7	9·4	6·7–12·6
3	Female	Ethnic minority	41–60	No qualification	56	11·8	8·9–15·2
4	Male	Ethnic majority	25–40	No qualification	15	11·9	8·9–15·7
5	Female	Ethnic majority	25–40	No qualification	37	12·1	9·2–15·5
**5 highest**							
44	Female	Ethnic minority	60+	Higher degree	79	33·6	27·8–39·4
45	Male	Ethnic minority	60+	Higher degree	95	34·6	29·2–40·5
46	Female	Ethnic majority	41–60	Higher degree	1465	35·0	32·0–38·1
47	Male	Ethnic majority	60+	Higher degree	784	39·3	36·2–42·1
48	Female	Ethnic majority	60+	Higher degree	1005	40·2	36·7–43·8

FVC, fruit and vegetable consumption.

## Discussion

This study aimed to explore intersectional inequalities in FVC in the UK based on educational level, sex, ethnicity and age, using nationally representative data from Wave 9 (2017–2018) of the UKHLS. Our results showed that FVC in the UK is below the recommended levels regardless of social strata, as reported in previous studies using UKHLS data.^(39)^ Of our sample, 69·2 % did not meet the daily recommended FVC guidelines. The majority of between-strata variance could be explained by additive effects alone. Although clear inequalities in FVC were identified, we did not find evidence that people belonging to specific combinations of educational level, sex, ethnicity and age systematically experienced reinforced (dis)advantages beyond their additive effects. This implies that the unidimensional inequalities (e.g. based on educational level) influenced FVC of people in a similar way, independent of their sex, age or ethnicity.

Out of the social identity variables examined, educational level had the largest independent influence on FVC in the UK. This finding aligns with evidence from studies conducted in the Canadian context.^(40,41)^ For example, Doan et al.^(41)^ also conducted an intersectional analysis of FVC. They examined inequalities in FVC across intersections of educational level and other factors, several of which were closely related to SEP, including household income, receipt of income support, employment status and household food insecurity. Similarly, they identified educational level as the most influential predictor of FVC.

To reduce educational inequalities in FVC, it is important to identify what barriers people with a lower educational level experience in their FVC. Lower educational attainment is often associated with lower income and other indicators of socio-economic disadvantage.^(42)^ However, the strong influence of educational level, even after accounting for factors such as income,^(41)^ highlights the importance of mechanisms specifically tied to education. These may include differences in skills, knowledge, social norms, taste and processes of social distinction, in addition to cost-related aspects.^(40,41,43)^

Nonetheless, costs likely still play a substantial role in shaping FVC, since FV-richer diets are often more expensive,^(44–46)^ and households with lower income spend a higher percentage of total expenditure on food.^(47)^ Studies have shown that individuals from lower SEP backgrounds place more importance on price in food purchasing decisions than individuals from higher SEP backgrounds,^(15,48)^ highlighting the benefit of reducing cost-related barriers to FVC. In their intersectional analysis, Doan et al.^(41)^ specifically highlighted an elevated risk of low FVC among individuals at the intersection of a low educational level and household food insecurity. Potential strategies to reduce cost-related FVC barriers include subsidies and vouchers for local FV vendors, which have been shown to increase FVC among people in low-SEP communities in England.^(49)^ Further, in light of recent supply shortages of FV in the UK,^(50)^ additional measures need to be taken to ensure universal year-round access, especially in neighbourhoods in which predominantly people with a lower SEP reside.

The use of intersectionality as a theoretical framework for quantitative research can help researchers identify inequalities and injustices and better understand the social patterning of inequalities. Currently, MAIHDA is considered to be the gold standard for exploring quantitative intersectional health inequalities.^(35)^ MAIHDA has considerable advantages over conventional models in intersectional research, such as its ability to adjust estimates for sample size of smaller strata, reducing bias.^(33)^ A number of European studies have employed this method to conduct intersectional research into a range of other public health issues, such as mental health,^(51)^ smoking^(52)^ and heart disease.^(53)^ To our knowledge, only one previous study^(12)^ has examined intersectional inequalities in FVC or other dietary behaviours in the UK. This study suggested that educational inequalities in FVC may be shaped by factors such as social relationships and living situation.

This study has several limitations. Only one variable has been used to account for SEP. In the main analysis, educational level was used as the sole SEP indicator. To provide further insight into the role of alternative SEP indicators, Appendix A contains sensitivity analyses using income level instead of educational level. Although the sensitivity analyses reveal that our conclusions may be robust across these two SEP indicators, it must be noted that unidimensional indicators such as educational or income level do not capture all aspects of SEP. Second, collapsing eighteen ethnicities into a binary majority–minority variable may have obscured meaningful between-group differences and could explain the absence of detectable intersectional FVC disparities related to ethnicity. Third, while intersectional differences related to age were observed, the categorisation of age into three broad categories may have masked more nuanced age-related disparities. Furthermore, despite the breadth of the Understanding Society dataset, the format of its fruit and vegetable questions is subject to recall bias and may not provide precise estimates of portion sizes or preparation methods. Given these limitations, future research would benefit from re-examining intersectional inequalities in FVC in the UK using more recent data and expanding the representation of ethnic identities beyond binary categories. We recommend further investigation of the intersectional effects of educational level in combination with factors such as food insecurity and social relationships in relation to diet. Such quantitative intersectional studies have the potential to provide a more nuanced understanding of the contribution of social and structural inequalities to health disparities.

## Conclusion

The results of this study provide novel insights into inequalities in FVC in the UK. Inequalities were mainly explained by additive effects. The analysis detected social groups with a higher risk of unhealthy FVC, such as young people, ethnic minority individuals and males without educational qualification. Given the particularly low rates of FVC in the UK overall, and the increased risk among those who experience one or more additive effects, there appears to be a need for proportionate universal interventions to increase FVC, rather than selective preventive measures. Since FVC is notably low among those with lower educational levels, policies that improve access to fruits and vegetables in all neighbourhoods, with additional attention to neighbourhoods in which predominantly people with lower educational levels reside, seem justified.

## Supporting information

10.1017/S1368980026102328.sm001 Stehl et al. supplementary material Stehl et al. supplementary material

## Supplementary material

For supplementary material accompanying this paper visit https://doi.org/10.1017/S1368980026102328
