# Supplementary material for: Uncovering intersectional inequalities in fruit and vegetable consumption in the UK using Understanding Society data
Source: Public Health Nutr. 2026 Mar 26;29(1):e79. doi: 10.1017/S1368980026102328 (PMC13418405; doi:10.1017/S1368980026102328)
Supplement: Stehl et al. supplementary material [file S1368980026102328sup001.docx]

**Appendix A**

A sensitivity analysis was conducted using household income as the indicator of socioeconomic position (SEP). The analytic sample included 16,070 participants, with all other variables retained as in the primary analysis. Household income was measured as average monthly net income in British Pounds (£) in Wave 9 of the UKHLS. The variable was obtained as a continuous variable and categorized into four groups for this analysis: (1) > £4,000 (25.3%), (2) £2,500–£3,999 (27.6%), (3) £1,500–£2,499 (26.1%), and (4) < £1,500 (20.9%). The results from MAIHDA are presented in Tables A1 and A2.

The results show that individuals who are not in the highest income group have lower odds of meeting the recommended FVC than individuals from the highest income group. The findings indicate that replacing education as an indicator of SEP with income reduces the explained variance at the intersectional level from 6,7% to 4.6%. This implies that educational disparities are a more salient driver of intersectional inequalities in healthy eating than income levels.

**Table A1**. Results from MAIHDA for adults in the UK in 2018. Model 1 is a random intercepts model with individuals nested within their social strata. Model 2 is fully adjusted, with all social dimensions being included as fixed main effects. Only the associations between the individual social dimensions and FVC are presented here.

|  | Model 1 | | Model 2 | |
| --- | --- | --- | --- | --- |
|  | OR | [95% CI] | OR | [95% CI] |
| **Fixed Effects: Regression Coefficients** |  |  |  |  |
| Intercept | 0.40 | [0.35, 0.45] | 0.34 | [0.29, 0.40] |
| Sex |  |  |  |  |
| Male (Ref) |  |  | - |  |
| Female |  |  | 1.64 | [1.49, 1.79] |
| Ethnicity |  |  |  |  |
| Ethnic majority (Ref) |  |  | - |  |
| Ethnic minority |  |  | 0.87 | [0.81, 0.93] |
| Age |  |  |  |  |
| 25-40 years (Ref) |  |  | - |  |
| 41-60 years |  |  | 1.29 | [1.13, 1.48] |
| 60+ years |  |  | 1.66 | [1.45, 1.93] |
| Income |  |  |  |  |
| > £4,000 (Ref) |  |  | - |  |
| £2,500 - £3,999 |  |  | 0.83 | [0.73, 0.95] |
| £1,500 - £2,499 |  |  | 0.63 | [0.55, 0.73] |
| < £1,500 |  |  | 0.54 | [0.46, 0.64] |
| **Random Effects: Variances** |  |  |  |  |
| Stratum-Level | 0.16 | [0.09, 0.26] | 0.01 | [<0.01, 0.02] |
| **Summary Statistics** |  |  |  |  |
| Intra Class Correlation (ICC; 95% credible interval) | 4.6% | [2.8, 7.3] | 0.3% | [0.1, 0.6] |
| Proportional Change in Variance (PVC) |  |  | 94.0% |  |
| Area Under Receiver Operating Characteristic Curve (AUC) | 0.60 |  | 0.57 |  |

**Table A2.** Predicted incidence for sufficient fruit and vegetable consumption for adults in the UK in 2018 by intersectional strata. Based on model 1 multilevel regression analysis with participants at the first level and intersectional strata at the second level.

| Rank | Sex | Ethnicity | Age | Income | n | Predicted % meeting FVC recommendation | Approximate 95% CI |
| --- | --- | --- | --- | --- | --- | --- | --- |
| 5 lowest |  |  |  |  |  |  |  |
| 1 | Male | Ethnic minority | 25-40 | < £1500 | 30 | 14.2 | [11.4, 17.9] |
| 2 | Female | Ethnic minority | 25-40 | < £1500 | 72 | 14.3 | [11.4, 17.8] |
| 3 | Male | Ethnic majority | 25-40 | < £1500 | 109 | 15.4 | [12.6, 18.3] |
| 4 | Male | Ethnic minority | 25-40 | £1500 - £2499 | 63 | 15.4 | [12.3, 18.7] |
| 5 | Female | Ethnic minority | 25-40 | £1500 - £2499 | 135 | 15.6 | [12.9, 18.6] |
| 5 highest |  |  |  |  |  |  |  |
| 44 | Male | Ethnic majority | 60+ | £2500 - £3999 | 600 | 32.5 | [29.7, 35.5] |
| 45 | Female | Ethnic minority | 60+ | More than £4000 | 28 | 32.6 | [27.0, 37.4] |
| 46 | Male | Ethnic minority | 60+ | More than £4000 | 53 | 33.2 | [28.3, 38.5] |
| 47 | Male | Ethnic majority | 60+ | More than £4000 | 489 | 35.6 | [32.1, 38.8] |
| 48 | Female | Ethnic majority | 60+ | More than £4000 | 374 | 46.3 | [32.4, 40.1] |
